# Melatonin protects against arsenic trioxide-induced liver injury by the upregulation of Nrf2 expression through the activation of PI3K/AKT pathway

**DOI:** 10.18632/oncotarget.13931

**Published:** 2016-12-14

**Authors:** Yue Zhang, Zhengkai Wei, Weijian Liu, Jingjing Wang, Xuexiu He, Hailong Huang, Jiali Zhang, Zhengtao Yang

**Affiliations:** ^1^ College of Animal Science and Technology, Jilin Agricultural University, Changchun, People’s Republic of China; ^2^ Department of Clinical Veterinary Medicine, College of Veterinary Medicine, Jilin University, Changchun, Jilin, People’s Republic of China

**Keywords:** As2O3, melatonin, liver injury, Nrf-2, Pathology Section

## Abstract

Melatonin has been demonstrated to have anti-inflammatory and antioxidant effects. The aim of this study was to investigate the protective effects of melatonin on arsenic trioxide (As_2_O_3_)-induced toxicity in liver and oxidative stress in rats. The rats were injected with 3mg/kg As_2_O_3_ on alternate days and melatonin was given with an intraperitoneal injection (i.p.) 1 h before As_2_O_3_ treatment. On the 8th days, the rats were killed to determine liver histological injury, antioxidant activities and accumulation of arsenic in liver tissues. Our results showed that melatonin attenuated As_2_O_3_-induced hepatic pathological damage, liver parameters, liver ROS level, MDA level, and the retention of arsenic in liver tissues. Melatonin also improved the antioxidant enzymes SOD, GPX, and CAT activity induced by As_2_O_3_. Furthermore, melatonin improved the expression of Nrf2 and HO-1 In addition, melatonin was found to activate PI3K/AKT pathway. In conclusion, our results indicated that melatonin protected against As_2_O_3_-induced liver injury by inducing Nrf2/HO-1 expression via upregulation of PI3K/AKT pathway.

## INTRODUCTION

Arsenic is a well-known global groundwater contaminant [1]. It often causes serious negative health effects in humans [2]. Exposure of human to arsenic results in toxicity and usually causes various types of solid tumors, such as bladder, skin, and lung cancers [3, 4]. Liver is the major target organ for many toxic chemicals, such as arsenic [5]. The mechanisms underlying arsenic-induced hepatotoxicity are not précised understood. However, most previous studies demonstrated that oxidative stress is the major contributor in arsenic trioxide-induced liver injury [6]. Arsenic exposure resulted in the development of oxidative stress-induced liver damage both in rats and humans [7]. Many investigators have confirmed that antioxidants could attenuate hepatotoxicity induced by arsenic [8]. Nrf2 is known to be involved in the regulation of oxidative stress and activation of Nrf2 could induce the expression of HO-1. Recently, studies showed that activation of Nrf2 could attenuate As_2_O_3_-induced oxidative injury.

Melatonin, a secretory product of the pineal gland, has been reported to have anti-inflammatory and antioxidant effects [9]. Melatonin was found to inhibit neutrophil inflammation and mucus secretion in cigarette smoke-induced chronic obstructive pulmonary diseases [10]. Melatonin also inhibited LPS-induced inflammatory cytokines production in RAW264.7 cells [9]. Melatonin has been reported to reduce oxidative stress and preserve the fluidity of biological membranes [9]. Furthermore, melatonin has been reported to protect against metal-catalyzed molecular damage [11]. However, whether melatonin has protective effects on As_2_O_3_-induced toxicity in liver and oxidative stress in rats remains unclear. Therefore, we investigated the protective effects of melatonin against arsenic trioxide-induced liver injury.

## RESULTS

### Melatonin inhibits As2O3-induced ALT and AST levels in serum

**Figure 1:**
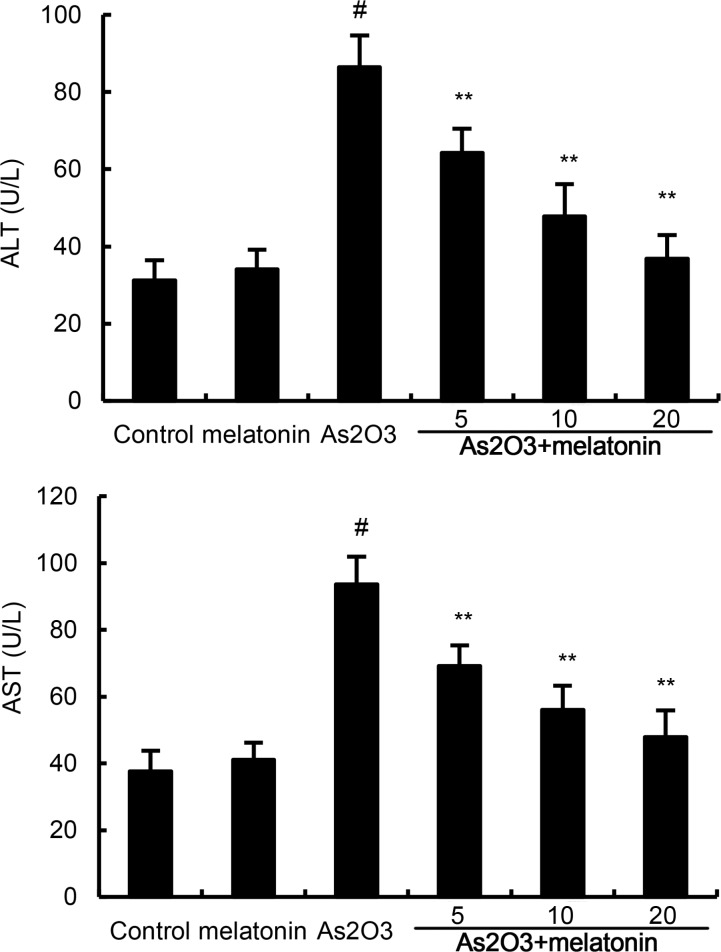
Effects of melatonin on As 2O3-induced ALT and AST levels. The values presented are the means ± SEM (n = 12 in each group). #p < 0.01 vs. control group, *p < 0.05 and **p < 0.01 vs. As2O3 group.

The effects of melatonin on As_2_O_3_-induced ALT and AST levels in serum were determined in this study. The results showed that As_2_O_3_ significantly up-regulated the production of ALT and AST. Treatment of melatonin dose-dependently inhibited As_2_O_3_-induced ALT and AST production (Figure 1).

### Melatonin inhibits As2O3-induced ROS and MDA levels

**Figure 2:**
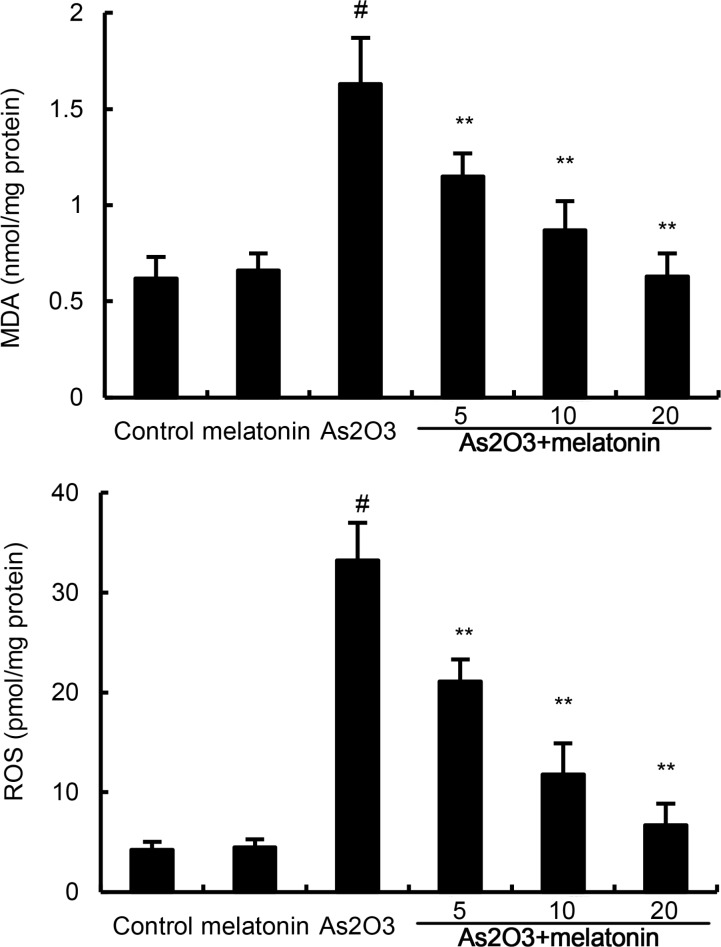
Effects of melatonin on As 2O3-induced ROS and MDA levels. The values presented are the means ± SEM (n = 12 in each group). #p < 0.01 vs. control group, *p < 0.05 and **p < 0.01 vs. As2O3 group.

The effects of melatonin on As_2_O_3_-induced ROS and MDA levels were used to assess the antioxidant effects of melatonin. As shown in Figure 2, the levels of ROS and MDA in liver tissues were significantly increased after As_2_O_3_ treatment. However, the production of ROS and MDA were suppressed by melatonin in a dose-dependently manner (Figure 2).

### Melatonin up-regulated the activity of antioxidant enzymes SOD, GPX, and CAT

**Figure 3:**
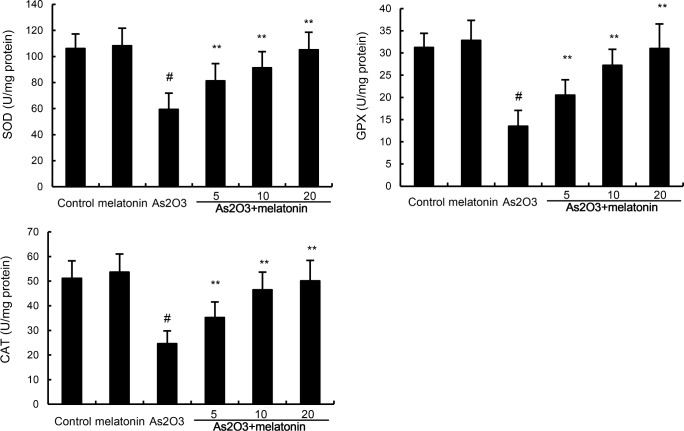
Effects of melatonin on As 2O3-induced antioxidant enzymes SOD, GPX, and CAT activity. The values presented are the mean ± SEM (n = 12 in each group). p# < 0.01 vs. control group, p* < 0.05, p** < 0.01 vs. As2O3 group.

Antioxidant enzymes SOD, GPX, and CAT were used to assess the antioxidant effects of melatonin. In this study, our results showed that the levels of SOD, GPX, and CAT decreased in As_2_O_3_-treated group when compared with the control group. However, melatonin up-regulated the production of SOD, GPX, and CAT inhibited by As_2_O_3_ (Figure 3).

### Melatonin attenuated As2O3-induced liver histopathologic changes

**Figure 4:**
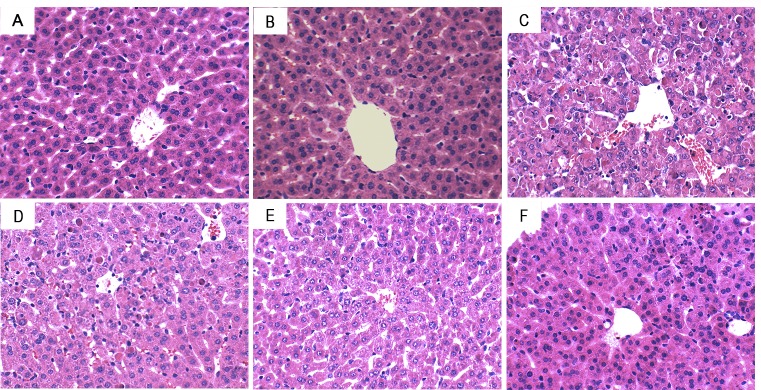
Effects of melatonin on As 2O3-induced liver histopathologic changes. Representative histological changes of liver obtained from mice of different groups. A. Control group, B. melatonin (20mg/kg) group, C. As2O3 group, D. melatonin (5mg/kg) + As2O3 (50 mg/kg) group, E. melatonin (10mg/kg) + As2O3 (50 mg/kg) group, F: melatonin (20mg/kg) + As2O3 (50 mg/kg) group (Hematoxylin and eosin staining, magnification 200×).

To assess the protective effects of melatonin on As_2_O_3_-induced liver injury, liver tissues histopathologic changes were detected. As shown in Figure 4, liver tissues of control and melatonin-treated groups showed a normal structure. Liver sections of As_2_O_3_-treated group showed severe pathologic changes, including extensive areas of portal inflammation, inflammatory cell infiltration and cellular necrosis. These pathological changes of liver sections were reduced by treatment of melatonin (Figure 4D, 4E, 4F).

### Melatonin attenuated the retention of arsenic in liver tissues

**Figure 5:**
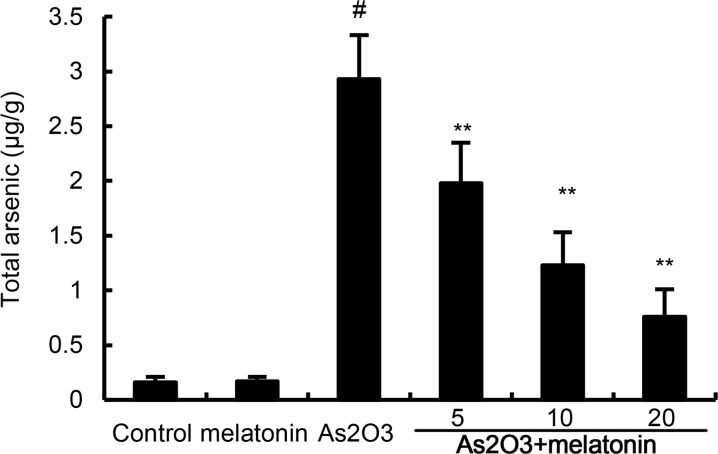
Effects of melatonin on As accumulation in liver tissues The values presented are the means ± SEM (n = 12 in each group). #p < 0.01 vs. control group, *p < 0.05 and **p < 0.01 vs. As2O3 group.

The effects of melatonin on the retention of arsenic in liver tissues were detected in this study. As shown in Figure 5, As_2_O_3_ resulted in a significant increase in the arsenic concentration of liver tissues. However, melatonin dose-dependently inhibited As_2_O_3_-induced accumulation of arsenic in liver tissues (Figure 5).

### Effects of melatonin on Nrf2 and HO-1 expression

**Figure 6:**
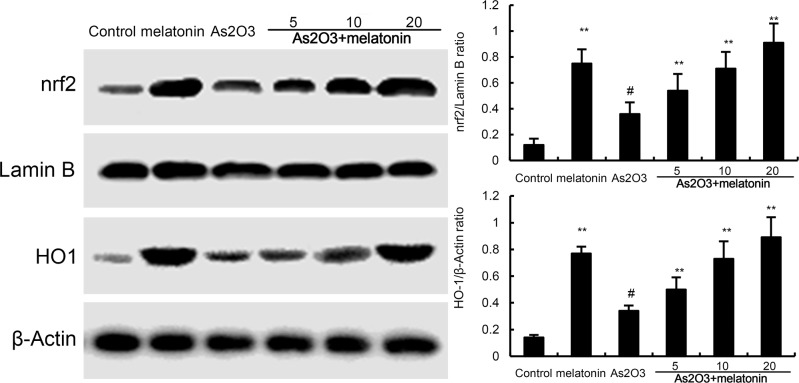
Effects of melatonin on Nrf2 and HO-1 expression The values presented are the means ± SEM (n = 12 in each group). #p < 0.01 vs. control group, *p < 0.05 and **p < 0.01 vs. As2O3 group.

To investigate the protective mechanism of melatonin, the effects of melatonin on Nrf2 and HO-1 expression were detected by western blotting. The results showed that nuclear translocation of Nrf2 and HO-1 expressions were increased by As_2_O_3._ These increases in Nrf2 and HO-1 expression were up-regulated by melatonin (Figure 6).

### Effects of melatonin on PI3K/AKT pathway

**Figure 7:**
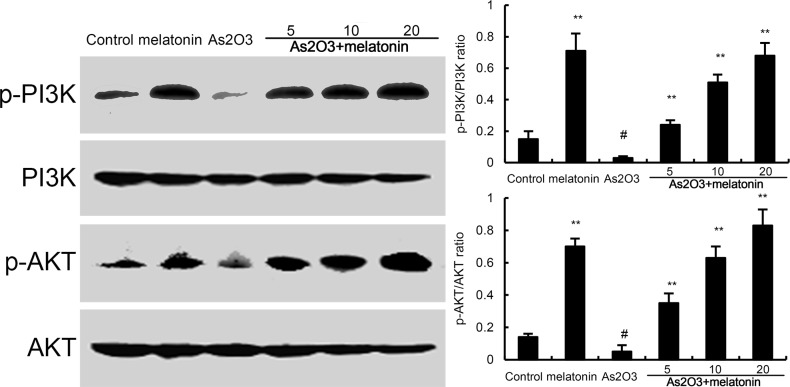
Effects of melatonin on PI3K/AKT phosphorylation The values presented are the means ± SEM (n = 12 in each group). #p < 0.01 vs. control group, *p < 0.05 and **p < 0.01 vs. As2O3 group.

The effects of melatonin on PI3K/AKT pathway were detected by western blotting. The results showed that As_2_O_3_ decreased the phosphorylation of PI3K and AKT. However, our results showed that melatonin upregulated the phosphorylation of PI3K and AKT (Figure 7).

## DISCUSSION

Arsenic, a well-known global groundwater contaminant, has been reported to cause severe health hazards [12]. In this study, we investigate the protective effects of melatonin on As_2_O_3_-induced liver injury in rats. Our results demonstrated that melatonin protected against As_2_O_3_-induced liver injury by reducing the retention of arsenic in liver tissues and activating of Nrf2 signaling pathway.

Previous studies showed that arsenic exposure resulted in the development of oxidative stress-induced liver damage both in rats and humans [13]. The generation of ROS after arsenic exposure has been reported to play a fundamental role in the induction of adverse health effects and disease [14]. MDA, an indicator of oxidant status, was used to assess the oxidative stress [15]. To determine oxidant stress, we measured ROS and MDA in liver tissues. Our results showed that treatment of As_2_O_3_ significantly increased the production of ROS and MDA in liver tissues. Melatonin dose-dependently inhibited As_2_O_3_-induced ROS and MDA production. In addition, melatonin was found to up-regulate the production of SOD, GPX, and CAT inhibited by As_2_O_3._ Recent studies showed that the antioxidants could be used as preventive and therapeutic agents against oxidative damage occurring during arsenic exposure [16, 17]. In this study, we found that melatonin attenuated As_2_O_3_-induced liver histopathologic changes. These results suggested that melatonin protected against As_2_O_3_-induced liver injury by inhibiting oxidative stress.

Nrf2 is a key transcription factor that binds and activates the antioxidant response element (ARE) in the promoters of many antioxidant genes [18, 19]. Nrf-2 is known to play a key role in orchestrating cellular antioxidant defenses and emerging data suggested that constitutive activation of Nrf2 had protective effects against As_2_O_3_-induced tissue injury [20, 21]. To investigate the antioxidant mechanism of melatonin, the effects of melatonin on Nrf-2 and HO-1 expression were detected by western blot analysis. The results showed that nuclear translocation of Nrf2 and HO-1 expressions were increased by melatonin. These increases in Nrf2 and HO-1 expressions were amplified by melatonin. These results suggested that melatonin exhibited antioxidant effects by activating Nrf2 signaling pathway. Previous research has demonstrated that the PI3K/AKT pathway plays a critical role in modulating Nrf2/HO-1 protein expression as an upstream signaling molecule [22]. In this study, we found melatonin upregulated the phosphorylation of PI3K and AKT. These results suggested that melatonin protected against As_2_O_3_-induced liver injury by inducing Nrf2/HO-1 expression via upregulation of PI3K/AKT pathway.

In conclusion, the results of this study showed that melatonin had a protective effect on As_2_O_3_-induced liver injury. Melatonin protected against As_2_O_3_-induced liver injury by the maintenance of redox homeostasis by inducing Nrf2/HO-1 expression via upregulation of PI3K/AKT pathway. These evidences suggested that melatonin had a potential application to treat As_2_O_3_-induced liver injury.

## MATERIALS AND METHODS

### Materials

As_2_O_3_ parenteral solution (10mg/ml) was purchased from Harbin Yida Pharmaceutical Company Ltd. (Harbin, China). Determination kits of ROS, GPX, SOD, CAT, and MDA were purchased from the Jiancheng Bioengineering Institute of Nanjing (Nanjing, Jiangsu, China). Antibodies specific for Nrf2, HO-1, Lamin B, β-actin, and horseradish peroxidase-conjugated (HRP) secondary antibodies were purchased from Santa Cruz Biotechnology (Autogen, Bioclear, UK). Melatonin (purity>98%) and all other chemicals were purchased from Sigma Aldrich (St. Louis, MO, USA).

### Animals

Male Wistar rats (8-9 week) were purchased from the Center of Experimental Animals of Jilin University (Changchun, China). The rats were kept in an environmentally controlled environment (temperature: 25 ± 2°C; humidity: 60 ± 5%, 12-h dark/light cycle). Water and pellet diets were supplied ad libitum. All animal experiments were approved by the NIH Guide for the Care and Use of Laboratory Animals.

### Experimental protocol

72 male Wistar rats were randomly divided into six groups and each group contains 12 rats: control group, melatonin treatment group, As_2_O_3_ group, and As_2_O_3_ + melatonin treatment group.

Control group: rats were treated with equal amount of 0.9% normal saline as a vehicle control.

Melatonin treatment group: rats were given by an intraperitoneal injection of melatonin (20mg/kg) once a day for 8 days.

As_2_O_3_ group: rats were administrated 3mg/kg As_2_O_3_ intravenous injection on alternate days for 4 days.

As_2_O_3_ + melatonin (5mg/kg) treatment group: rats were given by an intraperitoneal injection of melatonin (5mg/kg) once a day for 8 days.

As_2_O_3_ + melatonin (10mg/kg) treatment group: rats were given by an intraperitoneal injection of melatonin (10mg/kg) once a day for 8 days.

As_2_O_3_ + melatonin (20mg/kg) treatment group: rats were given by an intraperitoneal injection of melatonin (20mg/kg) once a day for 8 days.

On the 8th day, the rats were killed and the blood samples and livers from each group were collected for various biochemical analyses.

### Histological analysis

24 h after the last time of As_2_O_3_ treatment, the livers were collected. Liver tissues were fixed in 10% formalin, embedded in paraffin, and sliced. After hematoxylin and eosin (H&E) staining, histopathological changes were visualized with a microscope (Olympus, Japan).

### Measurement of oxidative stress and antioxidant enzymes in liver tissues

The levels of ROS, MDA, the antioxidant enzymes SOD, GPX, and CAT in liver tissues were detected by using commercial kits purchased from the Jiancheng Bioengineering Institute of Nanjing according to the manufacturer's instructions (Nanjing, Jiangsu, China).

### Measurement of ALT and AST

The ALT and AST levels in serum were detected by using test kits purchased from the Jiancheng Bioengineering Institute of Nanjing (Nanjing, Jiangsu, China) according to the manufacturer's instructions.

### Measurement of total arsenic in the liver

24 h after the last time of As_2_O_3_ treatment, the livers were collected. Then liver tissues were digested in HNO_3_-HCLO_4_ solution at 130°C for 2 days. After diluted with deionized water, the concentrations of arsenic in liver tissues were measured using atomic fluorescence spectrometry.

### Western blot analysis

Liver tissues were homogenized and total proteins were extracted using T-PER Tissue Protein Extraction Reagent Kit according to the manufacturer's instructions (Thermo Scientific, MA, USA). The protein concentration was determined through BCA method. 50 μg proteins per lane were separated by 12% SDS-PAGE gel. Then the proteins were transferred onto PVDF membranes. After blocking the nonspecific site with 5% nonfat dry milk, the membranes were probed with specific primary antibody at 4°C overnight. The membranes were incubated with secondary antibodies. Finally, the membranes were washed with PBS-T and then developed with the ECL Plus Western Blotting Detection System (Amersham Life Science, UK).

### Statistical analysis

Data are presented as mean±SEM of three separate experiments. Comparison between groups was made with ANOVA followed by Dunnett's test. The P< 0.05 was considered statistically significant.
